# Análise de um Registro de Base Populacional de Hospitalização por Infarto Agudo do Miocárdio

**DOI:** 10.36660/abc.20200611

**Published:** 2020-11-01

**Affiliations:** 1 Universidade Federal do Rio de Janeiro Rio de JaneiroRJ Brasil Universidade Federal do Rio de Janeiro, Rio de Janeiro, RJ - Brasil; 2 Universidade Severino Sombra Curso de Medicina VassourasRJ Brasil Universidade Severino Sombra - Curso de Medicina, Vassouras, RJ – Brasil

**Keywords:** Isquemia Miocárdica, Infarto do Miocárdio/mortalidade, Doenças Cardiovasculares/mortalidade, Morbidade, Hospitalização, Sistema Único de Saúde

A doença isquêmica do coração (DIC) é a principal causa de mortalidade no mundo, foi responsável por mais de 9 milhões de mortes no ano de 2016. O perfil de tendência nas taxas de mortalidade por DIC variou de acordo com o nível econômico dos países. Nações desenvolvidas experimentaram redução drástica nas taxas de mortalidade por DIC nos últimos anos, talvez atribuídas a maior foco na prevenção primária e melhor diagnóstico e tratamento. Porém, nos chamados países em desenvolvimento, a redução não foi tão acentuada, representando grande desafio para a saúde pública.^1^^,^^2^

Dentre as DICs a forma de apresentação de maior gravidade é o infarto agudo do miocárdio com supra desnivelamento do segmento ST (IAMCSST) que corresponde a cerca de um terço das apresentações das DICs, mas apresenta maior mortalidade quando comparado ao infarto agudo do miocárdio sem supra desnivelamento de segmento ST e com a angina instável.^3^^,^^4^

Estudar o comportamento dos casos de DIC, especialmente do IAMCSST com grande abrangência geográfica, populacional e temporal é essencial para melhorar a abordagem da maior causa de morte no mundo, justificando a realização e publicação do presente estudo.^5^ Vale uma crítica inicial ao estudo, pois na verdade se trata de um registro hospitalar e não obrigatoriamente populacional, pois se restringe aos dados colhidos a partir da internação de pacientes com IAMCSST em uma unidade hospitalar única e específica. Apesar da justificativa dos autores no fato do hospital de coleta dos dados ser a referência regional para a grande maioria dos pacientes com infarto que buscam inicialmente ou são posteriormente encaminhados para abordagem do IAM nesta instituição, não é possível estimar que a totalidade dos casos de IAMCSST da população alvo do estudo tenha sido tratada no centro de referência em questão. Algumas possibilidades devem ser levantadas, primeiro o fato de pacientes de outras localidades ao redor da região terem sido tratados no hospital do estudo ou até mesmo pacientes residentes da região terem apresentado IAMCSST e receberem tratamento em outro hospital da própria cidade ou de outro local.

A maior parte dos estudos epidemiológicos envolvendo o IAMCSST no Brasil aborda taxas de mortalidade,^6^^–^^8^ poucos dados de morbidade são analisados e divulgados. Um excelente indicador de morbidade, a taxa de internações, é abordada no presente estudo e muito bem explorada com divisão por faixa etária, busca de preditores e desfechos. Inclusive estas taxas de internações se demonstraram elevadas quando comparadas aos EUA ou a países europeus, mas não há disponibilidade de dados para comparação com taxas médias nacionais de internações por IAM e poucos dados populacionais confiáveis de outras regiões do Brasil podem ser encontrados. A divisão do sistema de saúde brasileiro em Sistema Único de Saúde (SUS) e sistema suplementar dificulta ainda mais a elaboração de taxas de internações nacionais por qualquer tipo de doença.^9^ A unidade hospitalar do estudo, conforme relatado, atende aos dois sistemas de saúde, pois afirma que 85% dos pacientes foram internados pelo SUS, o que nos faz supor que os outros 15% foram atendidos pelo sistema de saúde suplementar, portanto foi possível agrupar pacientes provenientes dos dois sistemas em um único estudo.

Podemos também caracterizar a cidade de Rio Grande, um município de porte médio com cerca de 200.000 habitantes, mas com elevado Índice de Desenvolvimento Humano Municipal (IDHM), 0,744, classificado como alto, entre 0,700 e 0,799.^10^

Os pontos positivos advindos do estudo epidemiológico em questão devem ser salientados. Envolveu mais de 500 pacientes provenientes de uma população de quase 200.000 habitantes. Foi um estudo prospectivo com longa duração, 4 anos, com seguimento dos pacientes por pelo menos um ano. Poucas perdas, menos de 10% por critérios de exclusão e sem perdas de recrutamento. Foi capaz de gerar dados de mortalidade, mas não se restringiu a isso, pois resultados com variáveis incluindo dados de morbidade e abordagem terapêutica também foram analisados e publicados. Estudos epidemiológicos com análise de variáveis capazes de medir adoecimento são essenciais para compreensão e busca de fatores para atingir a redução da incidência e da mortalidade da doença que mais causou morte no Brasil e no mundo nos últimos anos.
