# Supplementary material for: Análise de um Registro de Base Populacional de Hospitalização por Infarto Agudo do Miocárdio
Source: Arq Bras Cardiol. 2020 Nov 1;115(5):925–6. [Article in Portuguese] doi: 10.36660/abc.20200611 (PMC8452201; doi:10.36660/abc.20200611)
Supplement: Translation

# Translation: Analysis of a Population-Based Registry of Hospitalizations for Acute Myocardial Infarction

**Affiliations:** 1 Rio de JaneiroRJ Brazil Universidade Federal do Rio de Janeiro, Rio de Janeiro, RJ - Brazil; 2 VassourasRJ Brazil Universidade Severino Sombra - Curso de Medicina, Vassouras, RJ – Brazil

**Keywords:** Myocardial Ischemia, Myocardial Infarction/mortality, cardiovascular Diseases/mortality, Morbidity, Hospitalization, Unified Health System

Ischemic heart disease (IHD) is the leading cause of mortality in the world, being responsible for more than 9 million deaths in 2016. The trend profile of IHD mortality rates varied according to the economic level of countries. Developed nations have experienced a dramatic reduction in IHD mortality rates in recent years, perhaps attributed to a greater focus on primary prevention and better diagnosis and treatment. However, in the so-called developing countries, the reduction was not so accentuated, representing a major challenge for public health.^1^^,^^2^

Among the IHDs, the most severe form of presentation is acute myocardial infarction with ST-segment elevation (STEMI), which corresponds to approximately one-third of IHD presentations but has higher mortality when compared to non-ST-segment elevation myocardial infarction and unstable angina.^3^^,^^4^

Studying the behavior of IHD cases, especially of STEMI with wide geographical, population and temporal coverage, is essential to improve the approach to the biggest cause of death worldwide, justifying the performing and publication of the present study.^5^ An initial criticism of the study is worthwhile, as it is actually a hospital record and not necessarily a population record, as it is restricted to data collected from the admission of patients with STEMI to a single and specific hospital unit. Despite the authors' justification for the fact that the data collection hospital is the regional reference for the vast majority of patients with infarction who initially seek or are subsequently referred to this institution due to AMI, it is not possible to estimate that all cases of STEMI in the target population of the study has been treated at the study referral center. Some possibilities must be raised; first, the fact that patients from other locations around the region were treated at the study hospital or even the fact that patients who lived in the region had STEMI and received treatment at another hospital in the city or elsewhere.

Most epidemiological studies involving STEMI in Brazil address mortality rates,^6^^–^^8^ and few data on morbidity are analyzed and disclosed. An excellent indicator of morbidity, the rate of hospitalizations, is addressed in the present study and very well explored with divisions by age group, search for predictors and outcomes. Also, these hospitalization rates have shown to be high when compared to those in the USA or European countries, but there are no data available for comparison with the national average rates of hospitalizations for AMI and few reliable population data from other regions of Brazil can be found. The division of the Brazilian health system into the Unified Health System (SUS) and the supplementary health system makes it even more difficult to establish national hospitalization rates for any type of disease.^9^ The hospital unit of the study, as reported, treats patients from both health systems, as it states that 85% of patients were hospitalized through SUS, which makes us assume that the other 15% were treated through the supplementary health system, so it was possible to group patients from both systems in a single study.

We can also characterize the city of Rio Grande, a medium-sized municipality with around 200,000 inhabitants, but with an elevated Municipal Human Development Index (MHDI) of 0.744, classified as high, between 0.700 and 0.799.^10^

The positive points arising from the epidemiological study should be highlighted. It involved more than 500 patients from a population of almost 200,000 inhabitants. It was a prospective study with a long duration, 4 years, with follow-up of patients for at least one year. There were few losses, less than 10% by the exclusion criteria, and no recruitment losses. The study was able to generate mortality data, but it was not restricted to that, since results with variables including data on morbidity and therapeutic approach were also analyzed and published. Epidemiological studies with analysis of variables capable of measuring illness are essential to understand and search for factors aimed at achieving the reduction in the incidence and mortality of the disease that caused the most deaths in Brazil and worldwide in recent years.
